# Supplementary figures and images for: Cargoes move from cis to trans-Golgi compartments and concentrate in the TGN before exiting
Source: EMBO Rep. 2025 Sep 3;26(19):4742–65. doi: 10.1038/s44319-025-00548-9 (PMC12508209; doi:10.1038/s44319-025-00548-9)

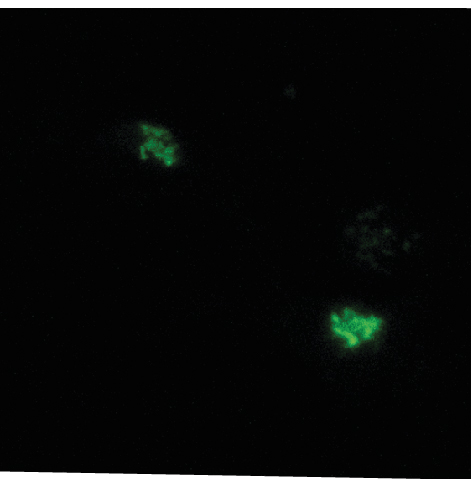

Supplement: Supplementary file 3 — Source data Fig. 1 [file 44319_2025_548_MOESM3_ESM.zip › Fig1/Fig1B_iFRAPt0.jpg]

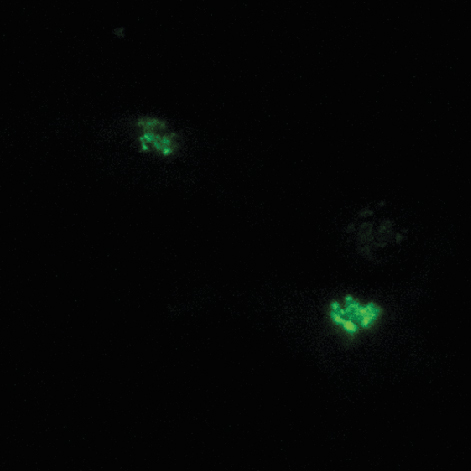

Supplement: Supplementary file 3 — Source data Fig. 1 [file 44319_2025_548_MOESM3_ESM.zip › Fig1/Fig1B_iFRAPt10.jpg]

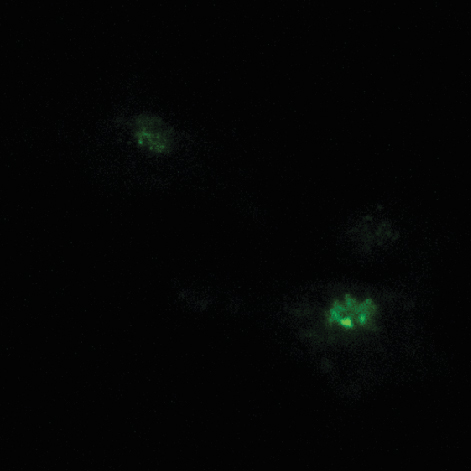

Supplement: Supplementary file 3 — Source data Fig. 1 [file 44319_2025_548_MOESM3_ESM.zip › Fig1/Fig1B_iFRAPt20.jpg]

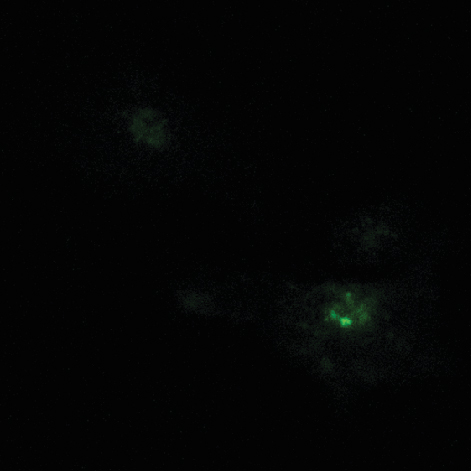

Supplement: Supplementary file 3 — Source data Fig. 1 [file 44319_2025_548_MOESM3_ESM.zip › Fig1/Fig1B_iFRAPt30.jpg]

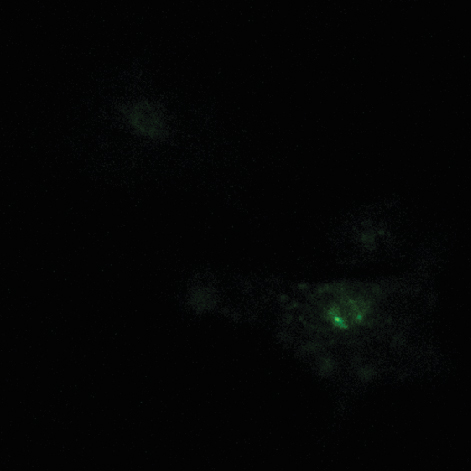

Supplement: Supplementary file 3 — Source data Fig. 1 [file 44319_2025_548_MOESM3_ESM.zip › Fig1/Fig1B_iFRAPt40.jpg]

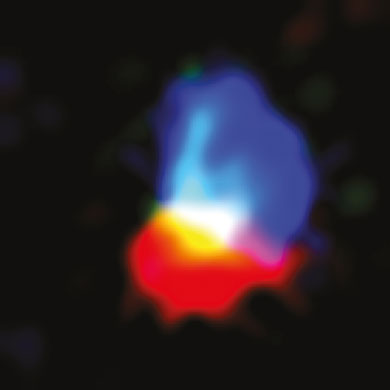

Supplement: Supplementary file 4 — Source data Fig. 2 [file 44319_2025_548_MOESM4_ESM.zip › Fig2/Fig2A_VSVG Rush 25min.jpg]

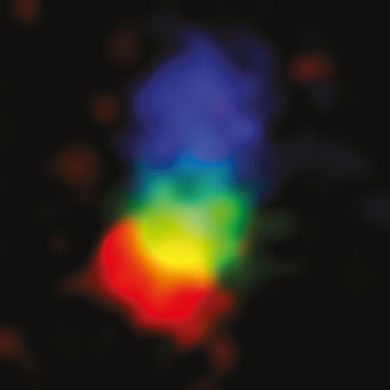

Supplement: Supplementary file 4 — Source data Fig. 2 [file 44319_2025_548_MOESM4_ESM.zip › Fig2/Fig2A_VSVG rush 15min.jpg]

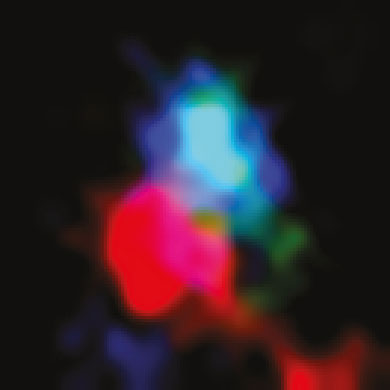

Supplement: Supplementary file 4 — Source data Fig. 2 [file 44319_2025_548_MOESM4_ESM.zip › Fig2/Fig2A_VSVG Rush 40min.jpg]

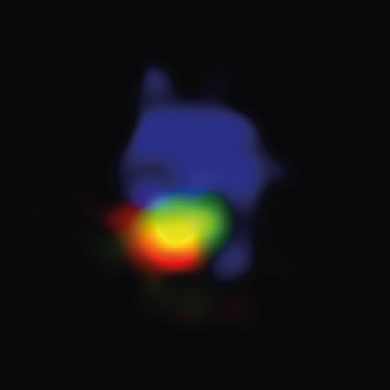

Supplement: Supplementary file 4 — Source data Fig. 2 [file 44319_2025_548_MOESM4_ESM.zip › Fig2/Fig2A_VSVG rush 5min.jpg]

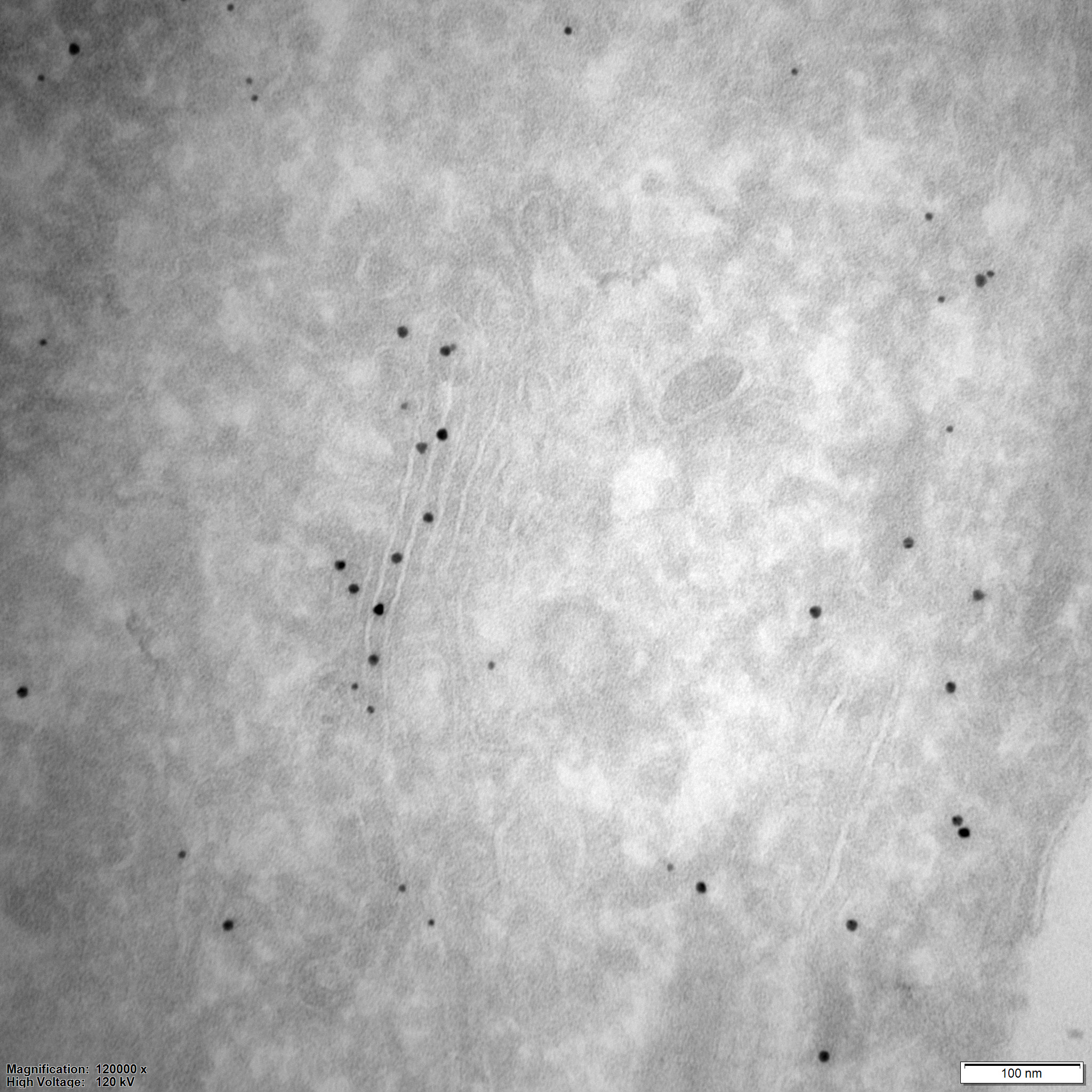

Supplement: Supplementary file 5 — Source data Fig. 3 [file 44319_2025_548_MOESM5_ESM.zip › Fig3/Fig3_5 min.tif]

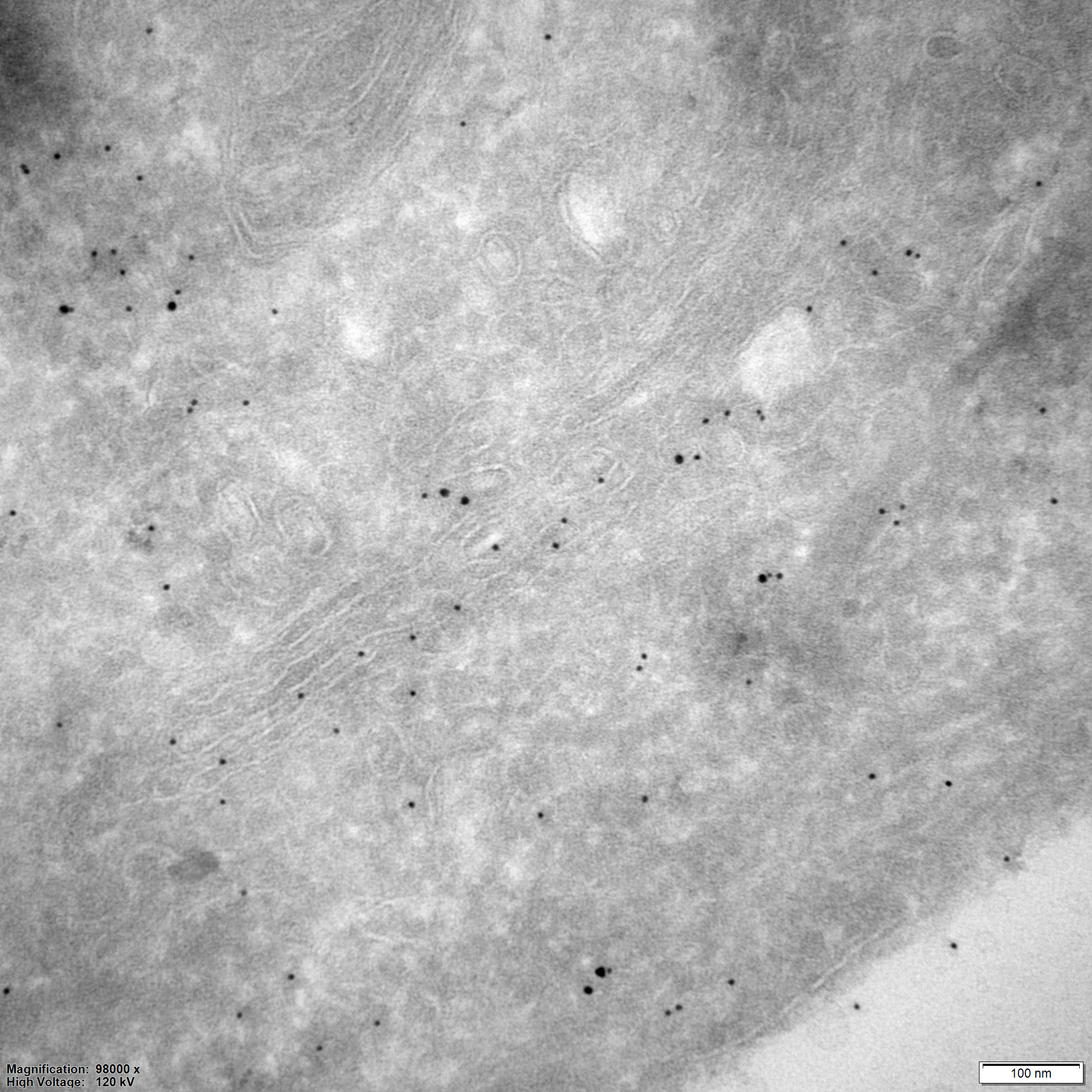

Supplement: Supplementary file 5 — Source data Fig. 3 [file 44319_2025_548_MOESM5_ESM.zip › Fig3/Fig3_25 min.tif]

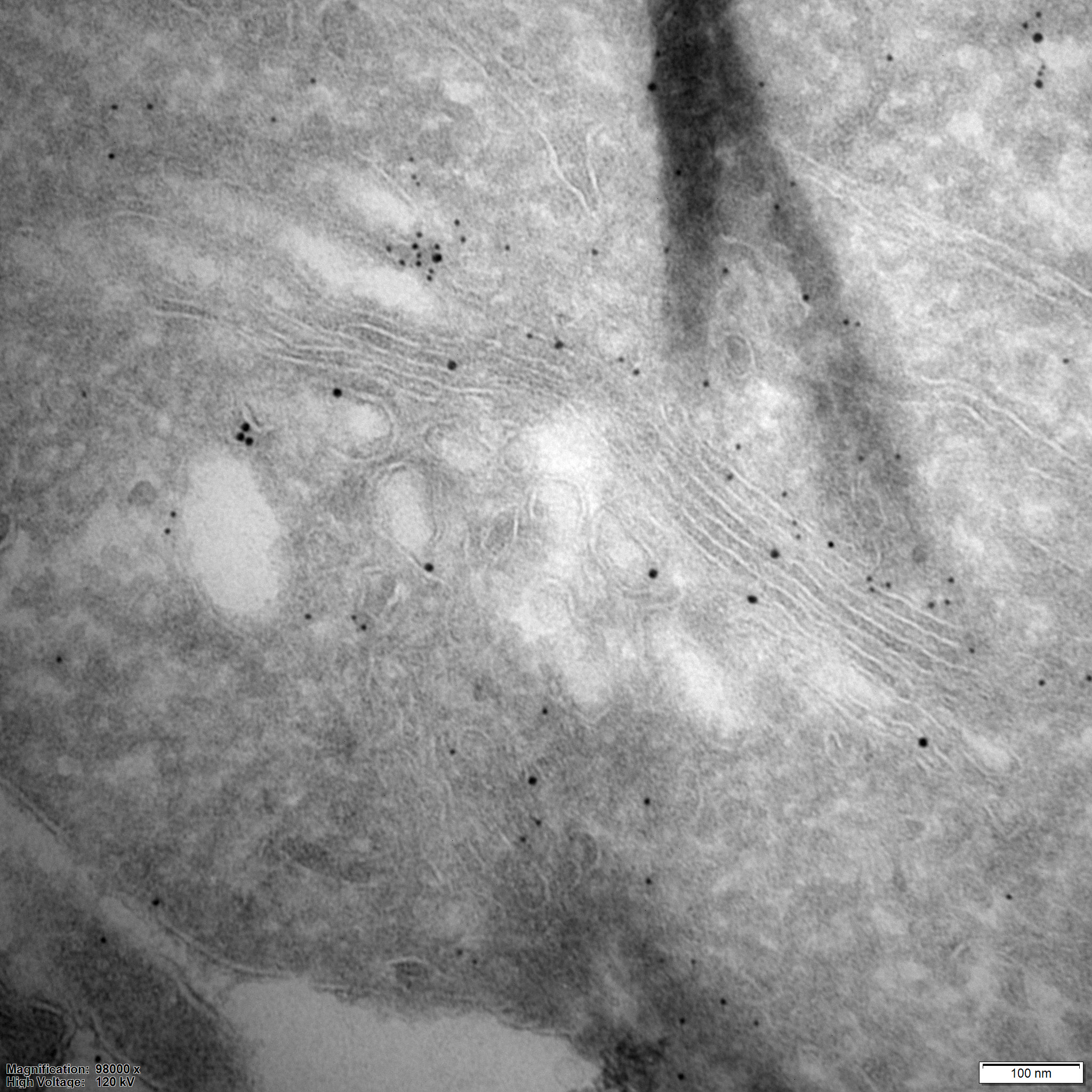

Supplement: Supplementary file 5 — Source data Fig. 3 [file 44319_2025_548_MOESM5_ESM.zip › Fig3/Fig3_40 min.tif]

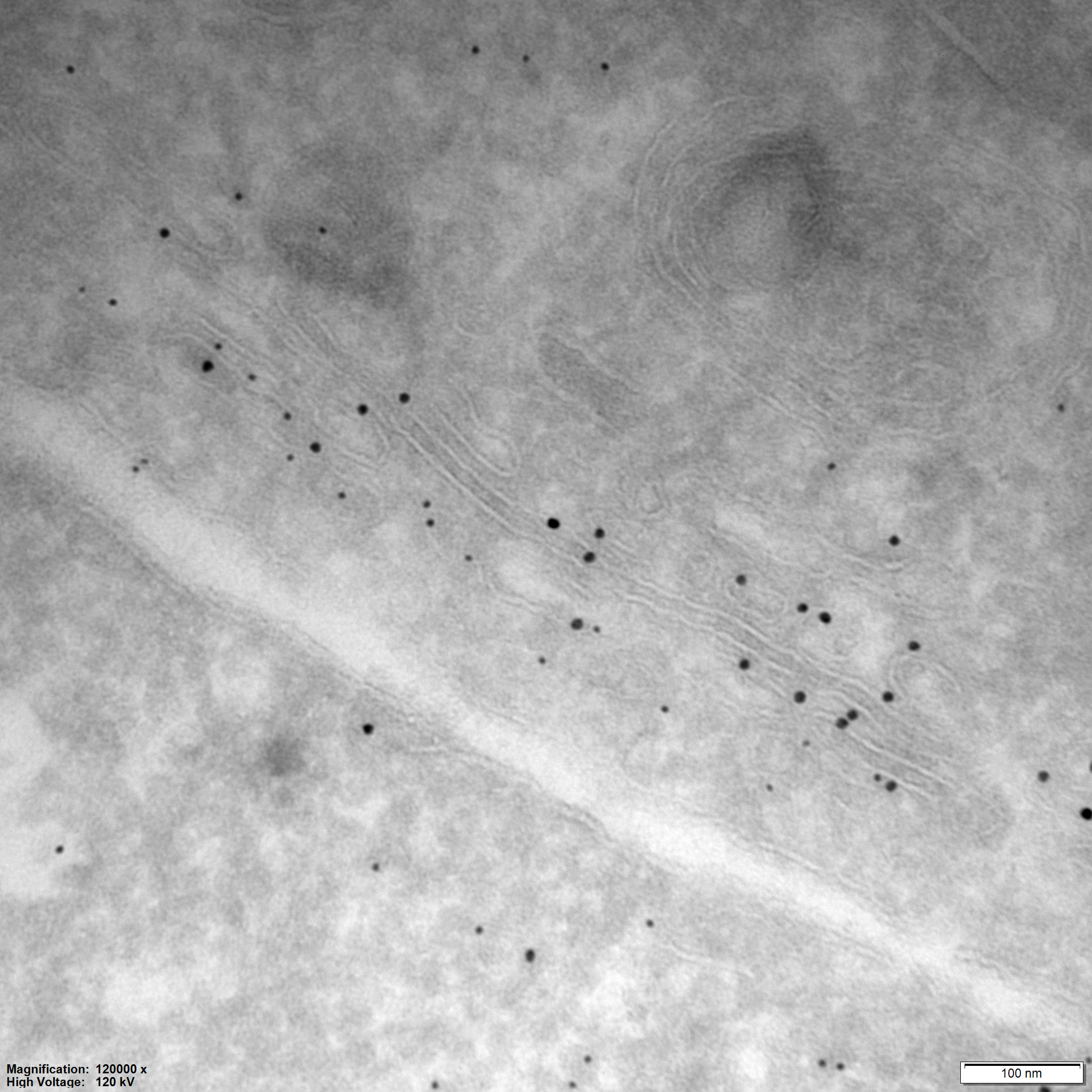

Supplement: Supplementary file 5 — Source data Fig. 3 [file 44319_2025_548_MOESM5_ESM.zip › Fig3/Fig3_15 min.tif]

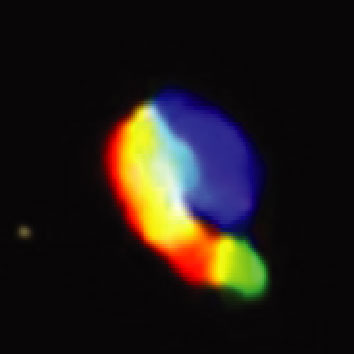

Supplement: Supplementary file 6 — Source data Fig. 4 [file 44319_2025_548_MOESM6_ESM.zip › Fig4/Fig4A_hgh rush 15min.jpg]

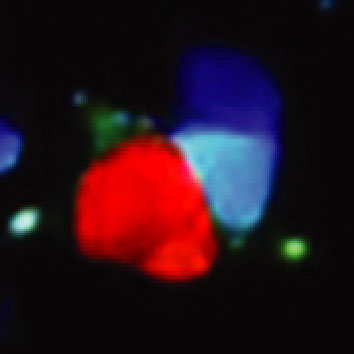

Supplement: Supplementary file 6 — Source data Fig. 4 [file 44319_2025_548_MOESM6_ESM.zip › Fig4/Fig4A_hgh rush 40min.jpg]

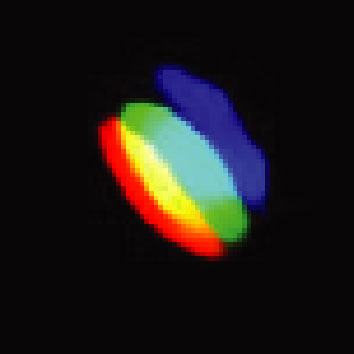

Supplement: Supplementary file 6 — Source data Fig. 4 [file 44319_2025_548_MOESM6_ESM.zip › Fig4/Fig4A_hgh rush 25min.jpg]

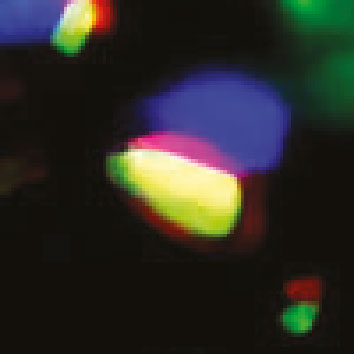

Supplement: Supplementary file 6 — Source data Fig. 4 [file 44319_2025_548_MOESM6_ESM.zip › Fig4/Fig4A_hgh rush 5min.jpg]

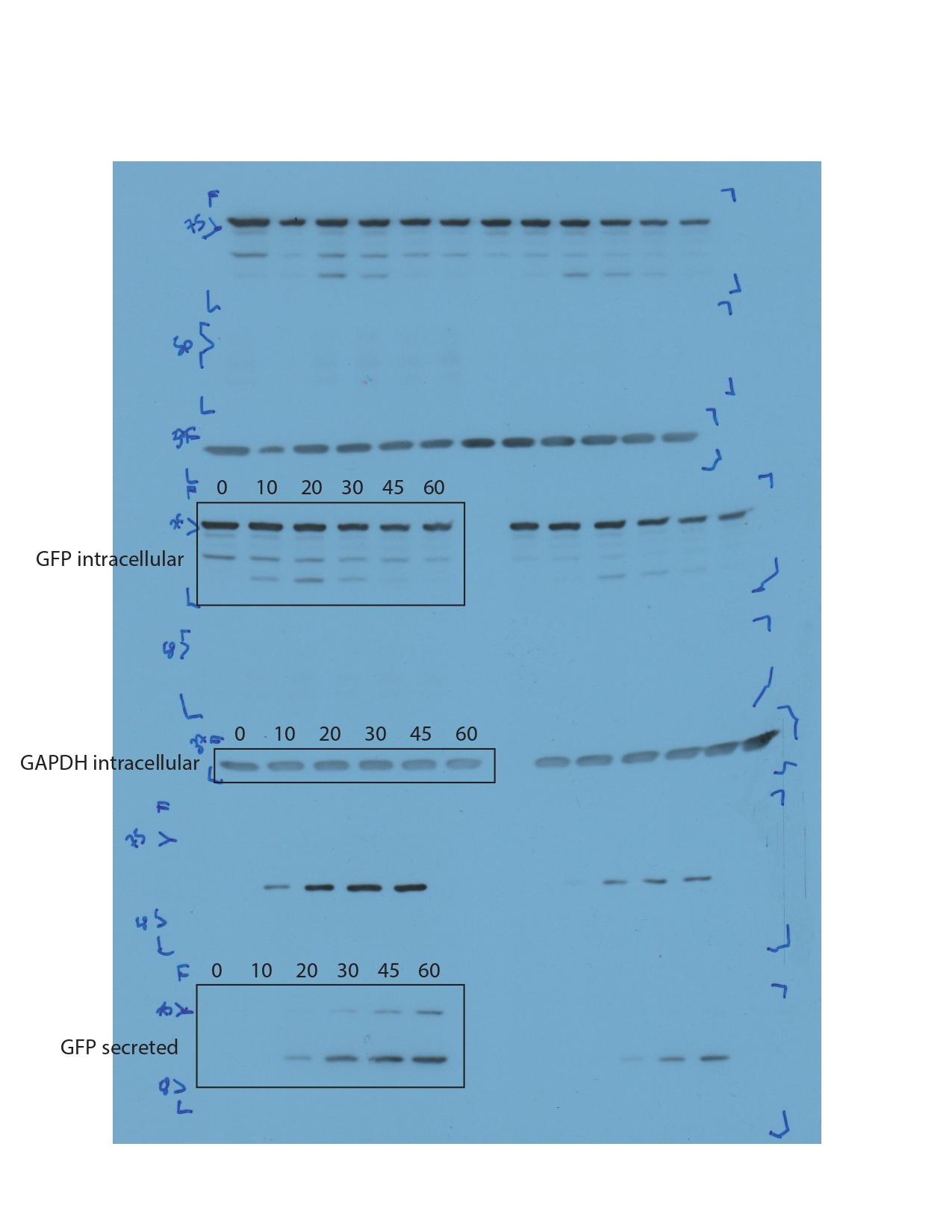

Supplement: Supplementary file 6 — Source data Fig. 4 [file 44319_2025_548_MOESM6_ESM.zip › Fig4/Fig4d.jpg]

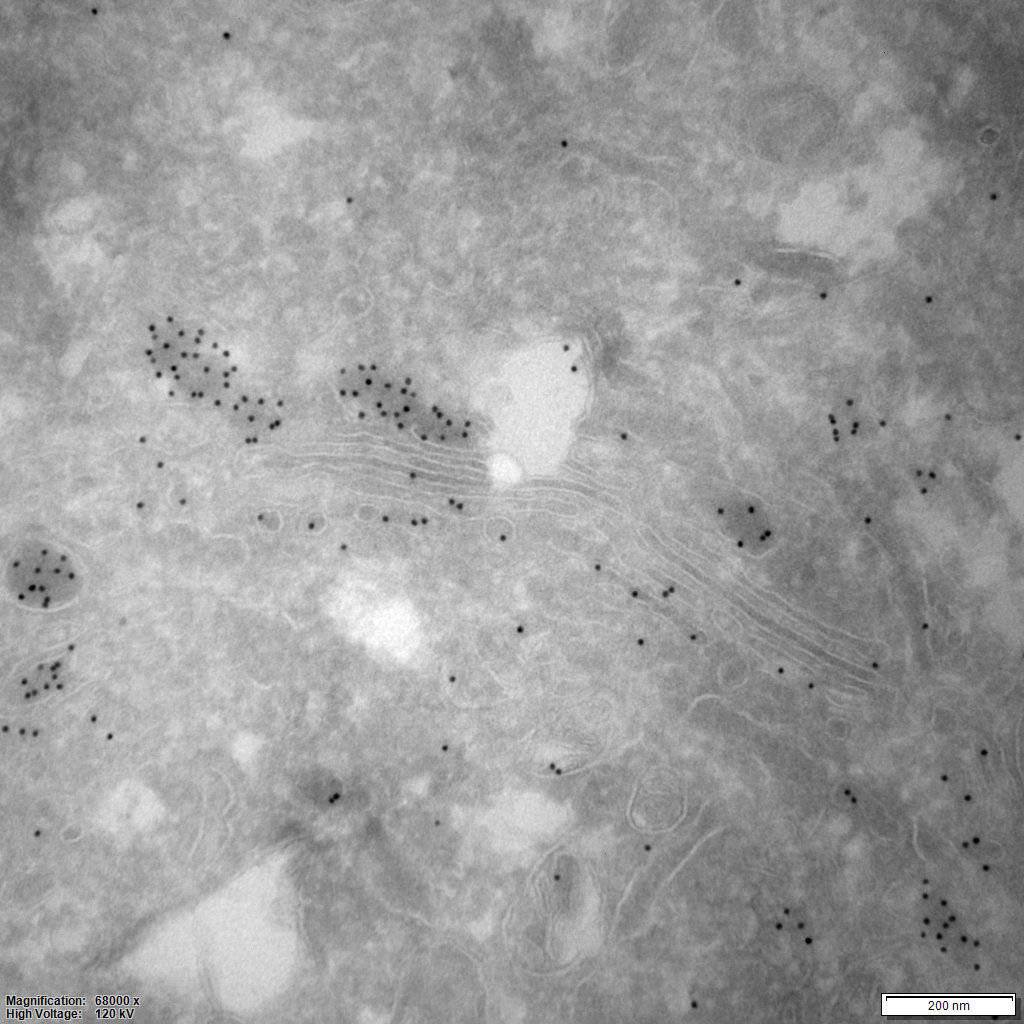

Supplement: Supplementary file 6 — Source data Fig. 4 [file 44319_2025_548_MOESM6_ESM.zip › Fig4/Fig4c.tif]

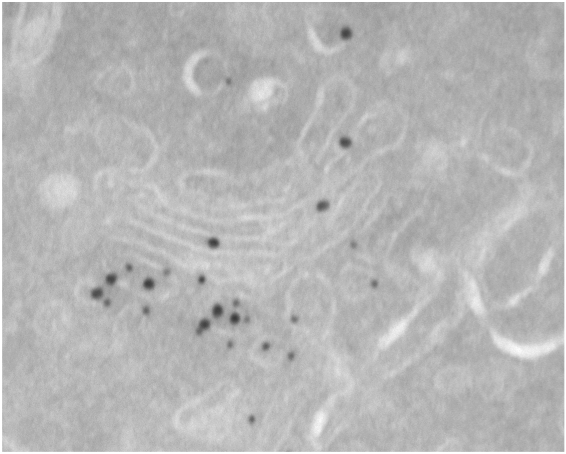

Supplement: Supplementary file 7 — Source data Fig. 5 [file 44319_2025_548_MOESM7_ESM.zip › Fig5/Fig5a.png]

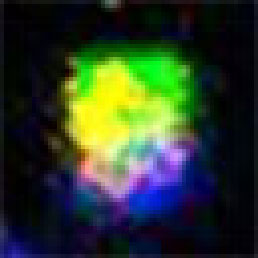

Supplement: Supplementary file 7 — Source data Fig. 5 [file 44319_2025_548_MOESM7_ESM.zip › Fig5/Fig5B/Fig5b VSVG Rush 60 min/VSVG Rush 60 min.jpg]

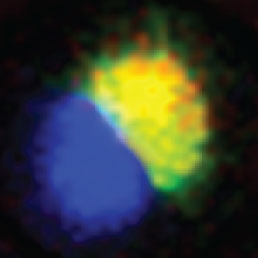

Supplement: Supplementary file 7 — Source data Fig. 5 [file 44319_2025_548_MOESM7_ESM.zip › Fig5/Fig5B/Fig5b GPI Rush 60 min/GPI Rush 60 min.jpg]

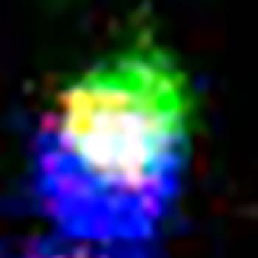

Supplement: Supplementary file 7 — Source data Fig. 5 [file 44319_2025_548_MOESM7_ESM.zip › Fig5/Fig5B/Fig5b LAMP1 Rush 60 min/LAMP1 Rush 60 min.jpg]

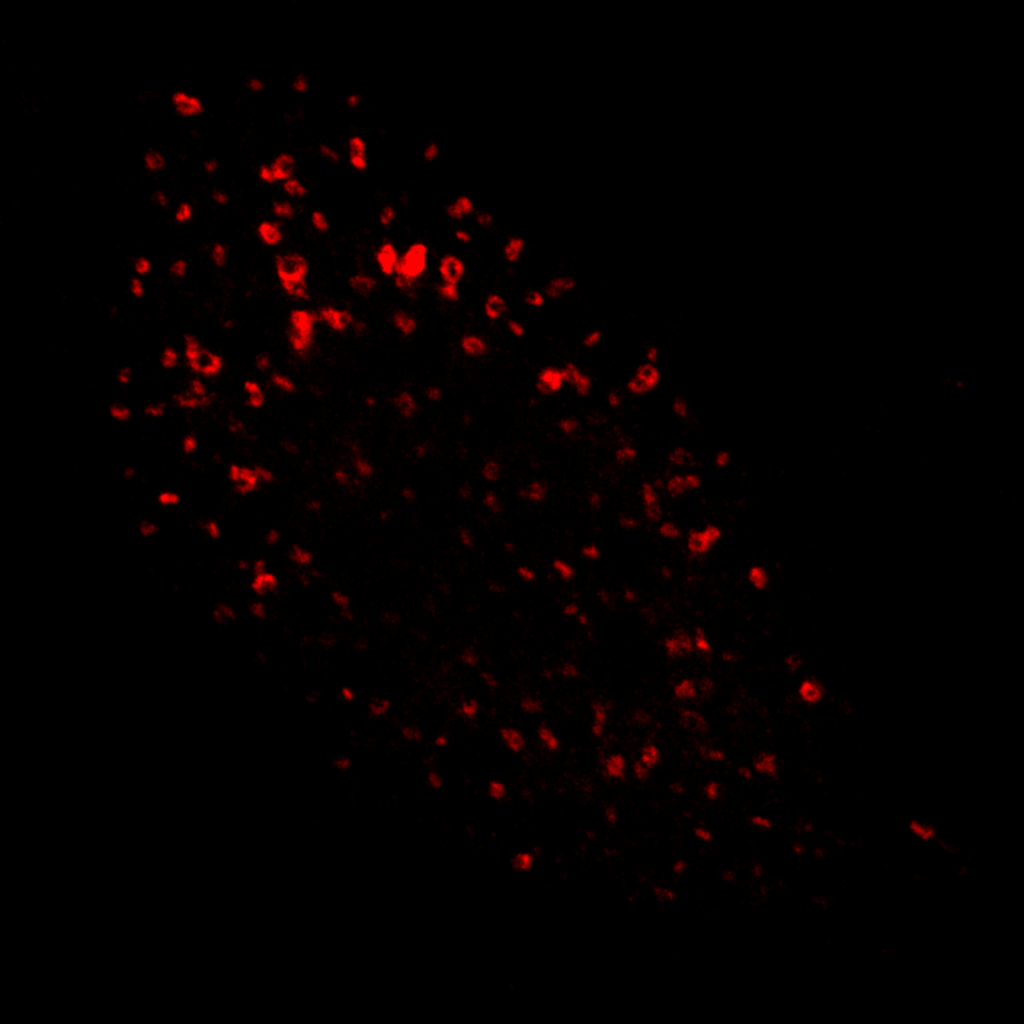

Supplement: Supplementary file 9 — Figure EV3 Source Data [file 44319_2025_548_MOESM9_ESM.zip › FigEV3/FigEV3A_3h giantin.tif]

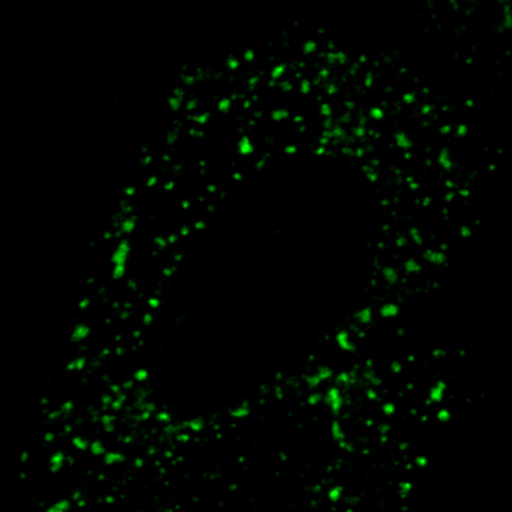

Supplement: Supplementary file 9 — Figure EV3 Source Data [file 44319_2025_548_MOESM9_ESM.zip › FigEV3/FIGEV3A_6h vsvg.tif]

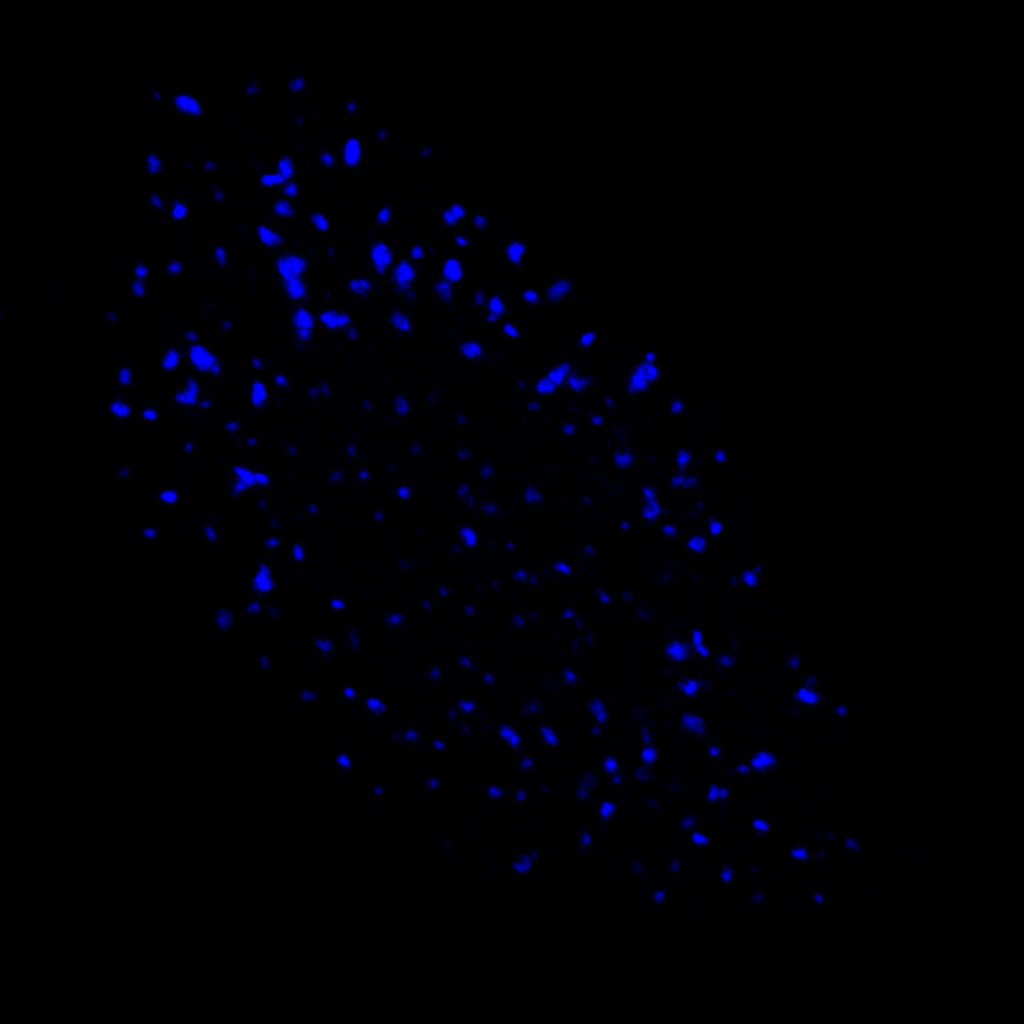

Supplement: Supplementary file 9 — Figure EV3 Source Data [file 44319_2025_548_MOESM9_ESM.zip › FigEV3/FIGEV3A_3h gm130.tif]

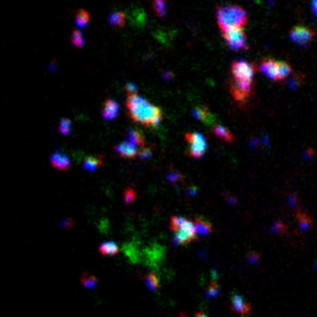

Supplement: Supplementary file 9 — Figure EV3 Source Data [file 44319_2025_548_MOESM9_ESM.zip › FigEV3/FIGEV3A_3h merge CROP.tif]

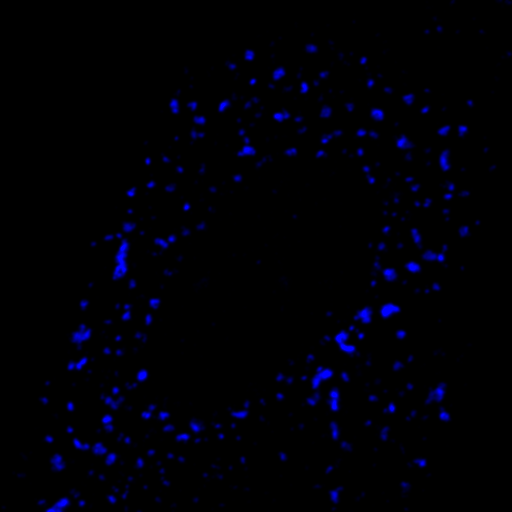

Supplement: Supplementary file 9 — Figure EV3 Source Data [file 44319_2025_548_MOESM9_ESM.zip › FigEV3/FIGEV3A_6h gm130.tif]

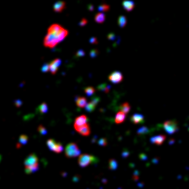

Supplement: Supplementary file 9 — Figure EV3 Source Data [file 44319_2025_548_MOESM9_ESM.zip › FigEV3/FIGEV3A_6h merge CROP.tif]

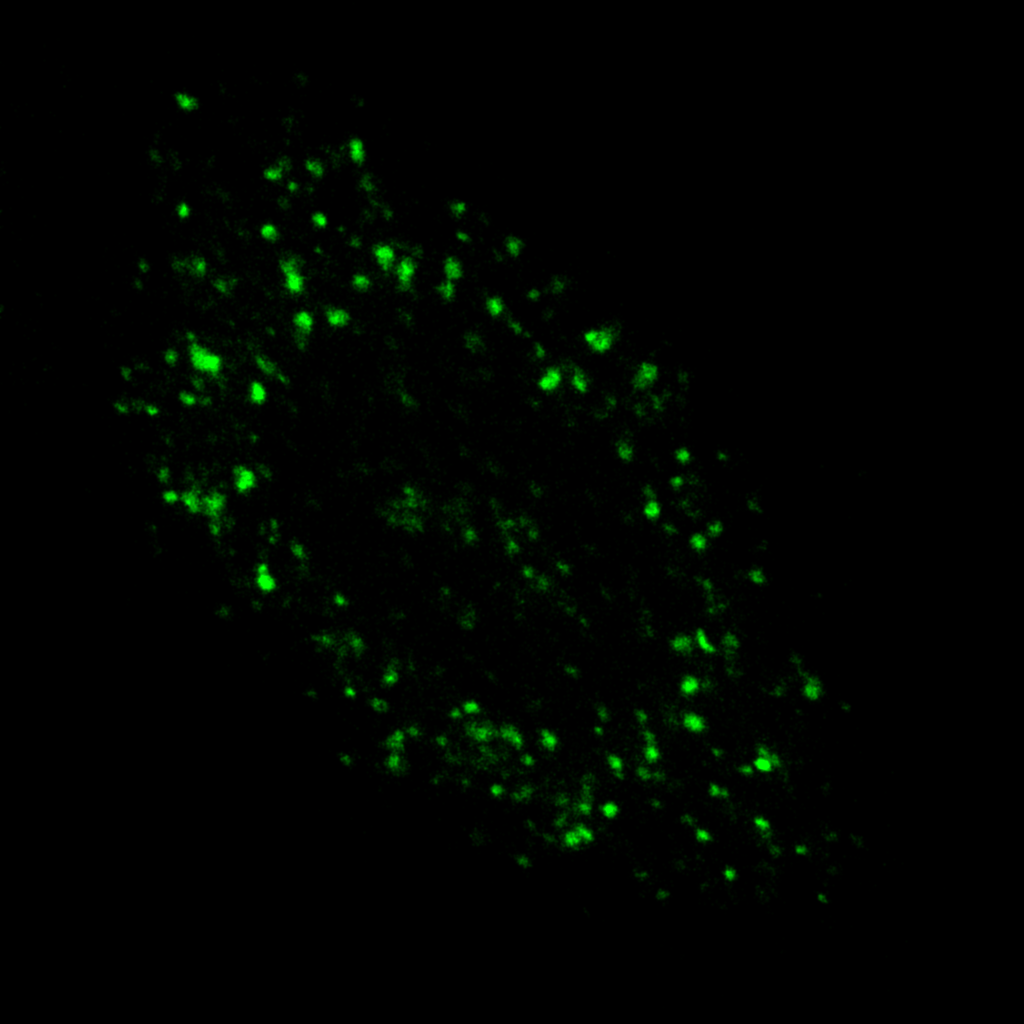

Supplement: Supplementary file 9 — Figure EV3 Source Data [file 44319_2025_548_MOESM9_ESM.zip › FigEV3/FIGEV3A_3h vsvg.tif]

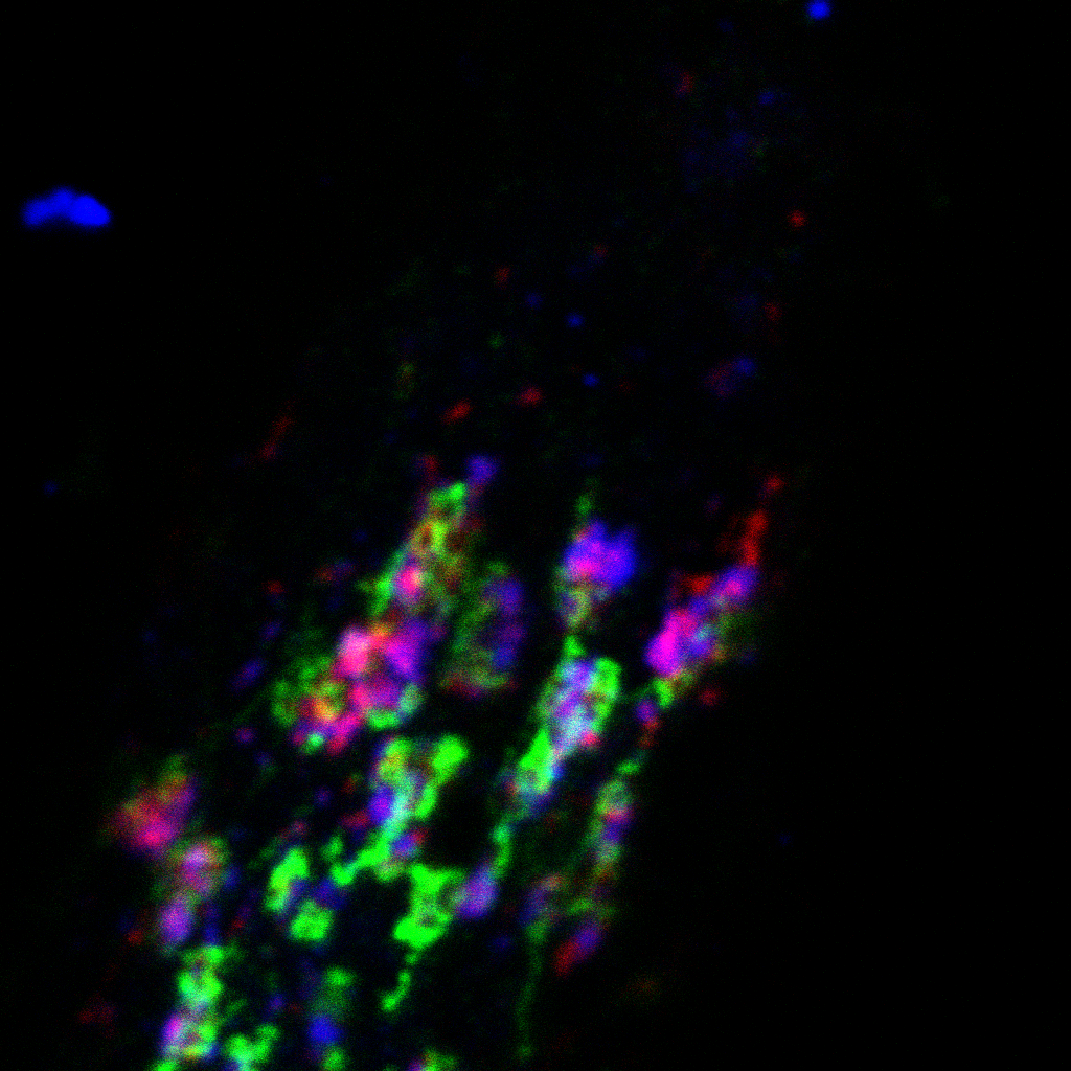

Supplement: Supplementary file 9 — Figure EV3 Source Data [file 44319_2025_548_MOESM9_ESM.zip › FigEV3/FigEV3d.tif]

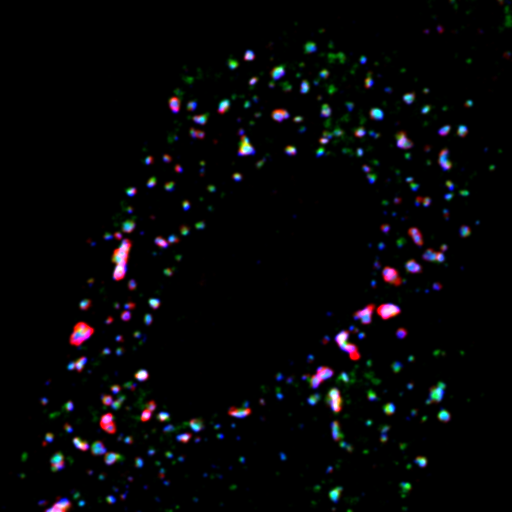

Supplement: Supplementary file 9 — Figure EV3 Source Data [file 44319_2025_548_MOESM9_ESM.zip › FigEV3/FIGEV3A_6h merge.tif]

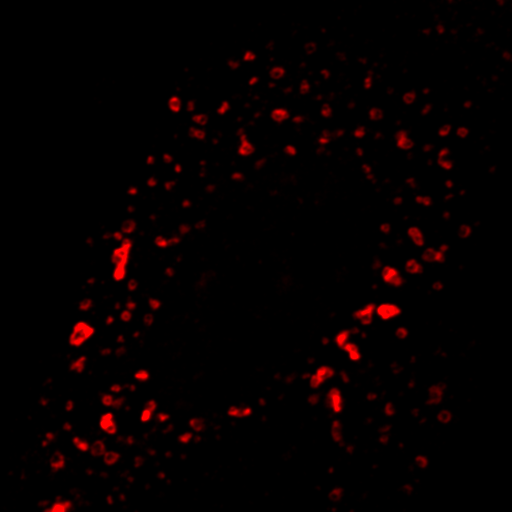

Supplement: Supplementary file 9 — Figure EV3 Source Data [file 44319_2025_548_MOESM9_ESM.zip › FigEV3/FIGEV3A_6h giantin.tif]

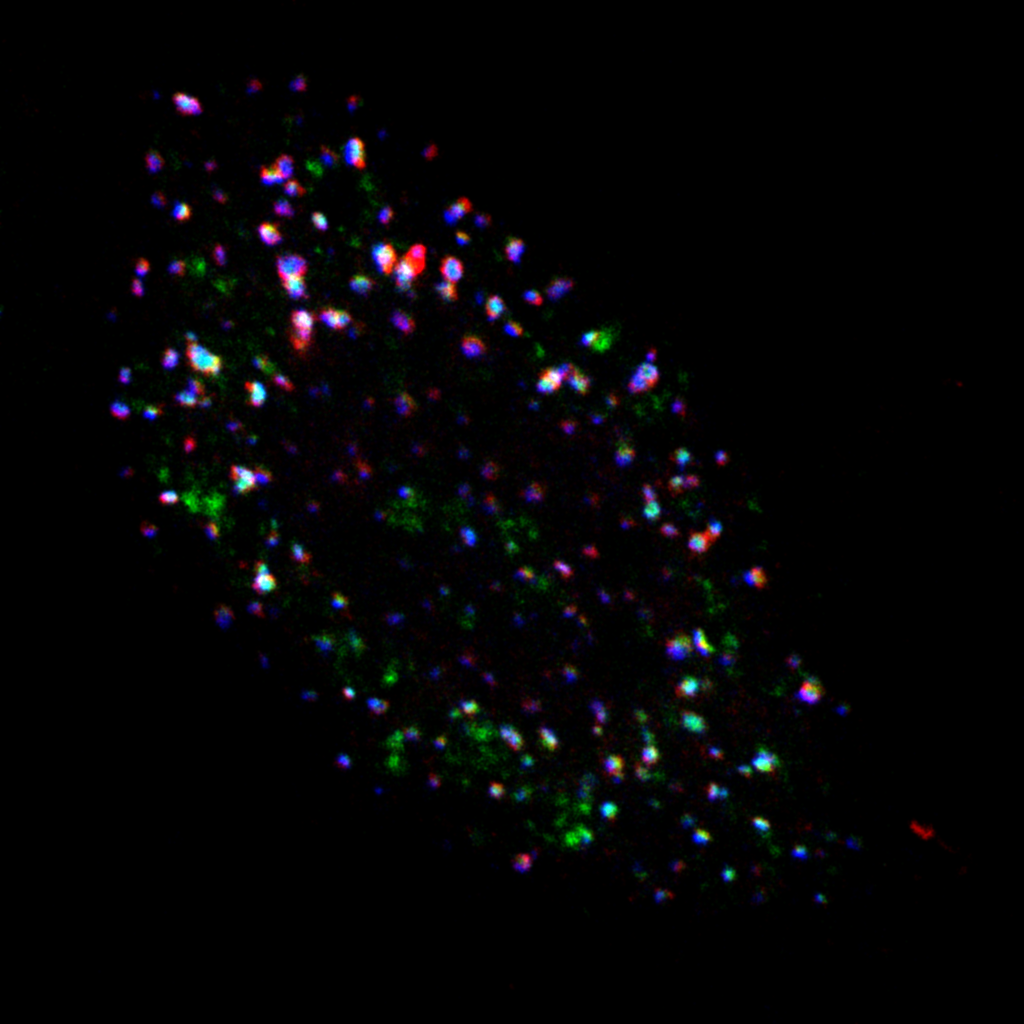

Supplement: Supplementary file 9 — Figure EV3 Source Data [file 44319_2025_548_MOESM9_ESM.zip › FigEV3/FIGEV3A_3h merge.tif]

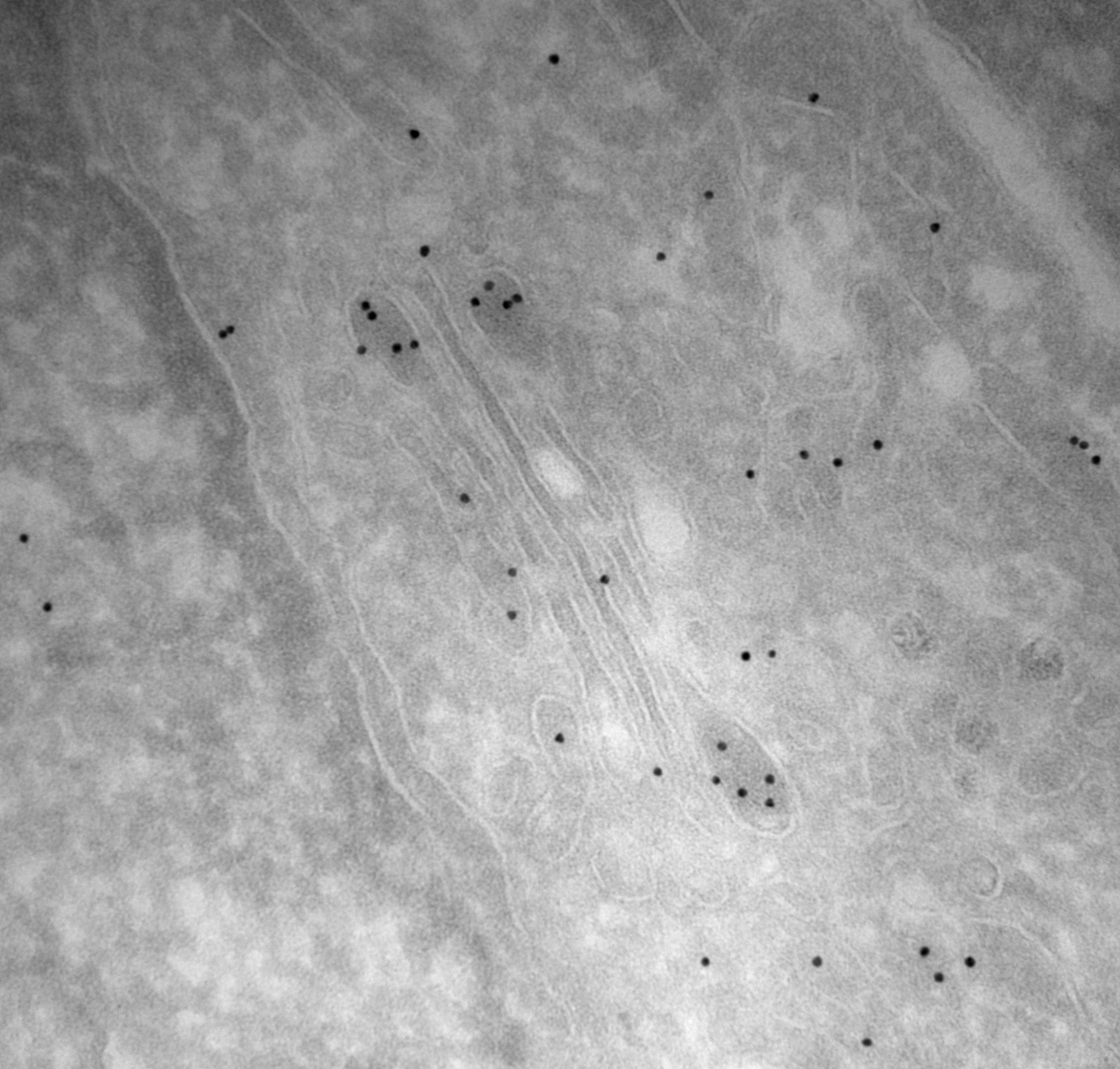

Supplement: Supplementary file 10 — Figure EV4 Source Data [file 44319_2025_548_MOESM10_ESM.zip › FigEV4/FigEV4f.tif]

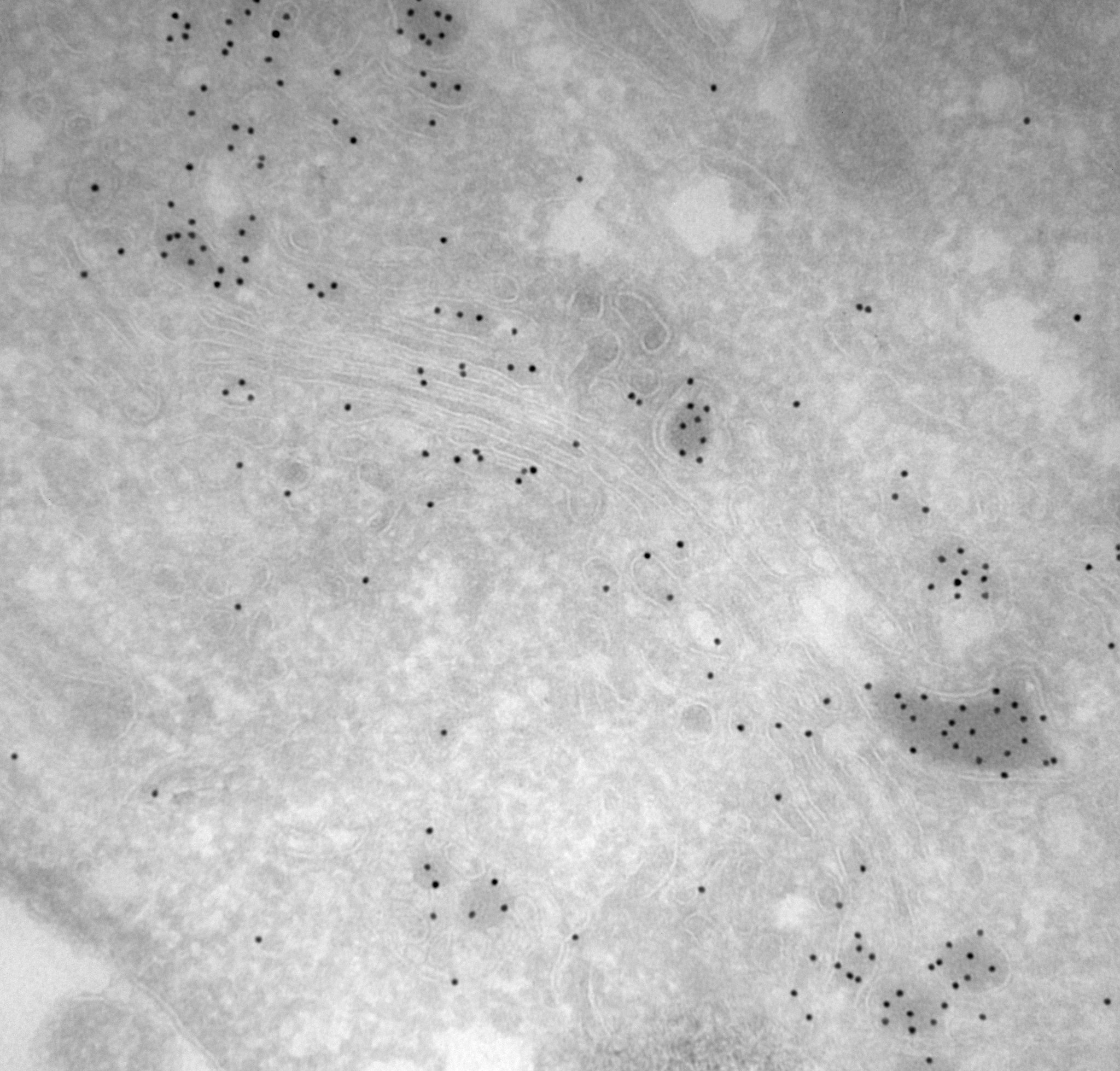

Supplement: Supplementary file 10 — Figure EV4 Source Data [file 44319_2025_548_MOESM10_ESM.zip › FigEV4/FigEV4e.tif]

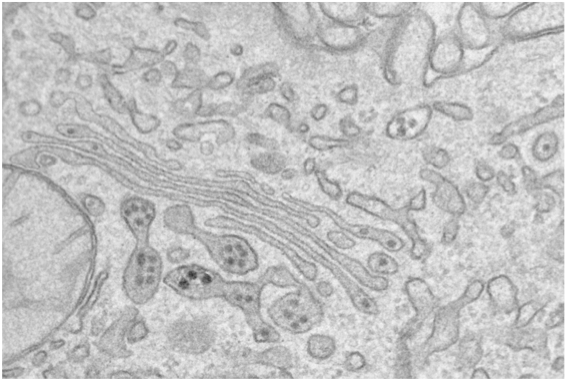

Supplement: Supplementary file 10 — Figure EV4 Source Data [file 44319_2025_548_MOESM10_ESM.zip › FigEV4/FigEV4d.png]

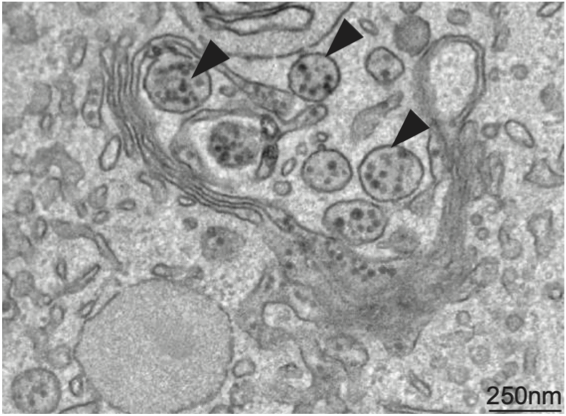

Supplement: Supplementary file 10 — Figure EV4 Source Data [file 44319_2025_548_MOESM10_ESM.zip › FigEV4/FigEV4A.png]

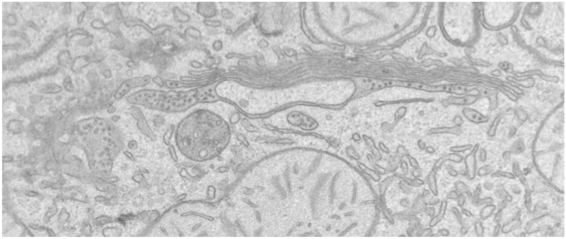

Supplement: Supplementary file 10 — Figure EV4 Source Data [file 44319_2025_548_MOESM10_ESM.zip › FigEV4/FigEV4b.png]

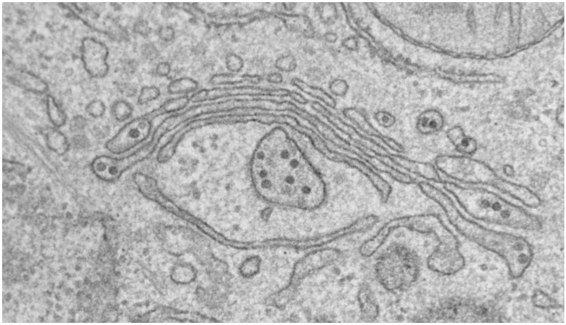

Supplement: Supplementary file 10 — Figure EV4 Source Data [file 44319_2025_548_MOESM10_ESM.zip › FigEV4/FigEV4c.png]
