# Supplementary material for: Cargoes move from cis to trans-Golgi compartments and concentrate in the TGN before exiting
Source: EMBO Rep. 2025 Sep 3;26(19):4742–65. doi: 10.1038/s44319-025-00548-9 (PMC12508209; doi:10.1038/s44319-025-00548-9)
Supplement: Supplementary file 11 — Expanded View Figures [file 44319_2025_548_MOESM11_ESM.pdf]

Expanded View Figures

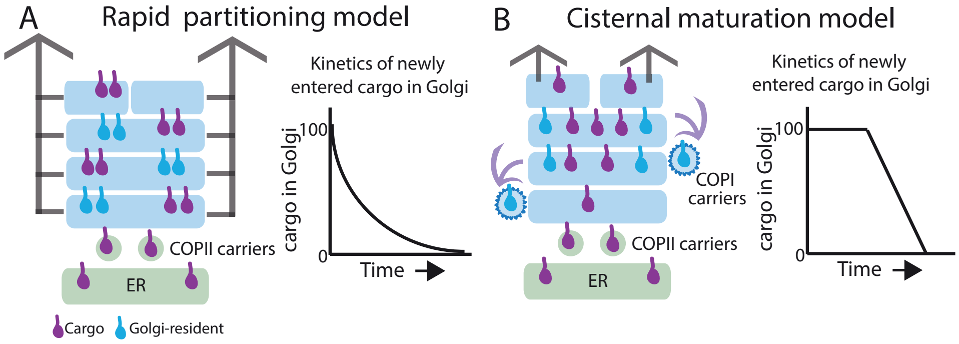

Figure EV1. Comparison of Rapid partitioning and Cisternal maturation models.

The comparison of rapid partitioning (**A**) and cisternal maturation model (**B**) of cargo transport through the Golgi. In the rapid partitioning model, the cargo that enters the Golgi distributes among the cisternae and leaves the Golgi from all the cisternae with no preferential site of exit. The amount of cargo exiting the Golgi depends on the amount of cargo present in the Golgi thus resulting in a monoexponential exit with no delay. Of note, in this model, there is no preferential exit of older or newly arrived cargoes from the Golgi. In the cisternal maturation model the cargoes remain in the cisterna that matures from *cis* to *trans*-Golgi. The cargoes thus arriving at the TGN (by the maturation of the *trans*-Golgi to TGN) is sorted into carriers that mature from the TGN to leave the Golgi. So, the kinetics of exit of newly arrived cargoes at the Golgi displays a flat or stationary phase that corresponds with the intra-Golgi transport and then linear kinetics of exit corresponding to the exit of the cargo. In this model, the older cargoes preferentially exit the Golgi first then followed by the newly arrived cargoes.

© The Author(s)

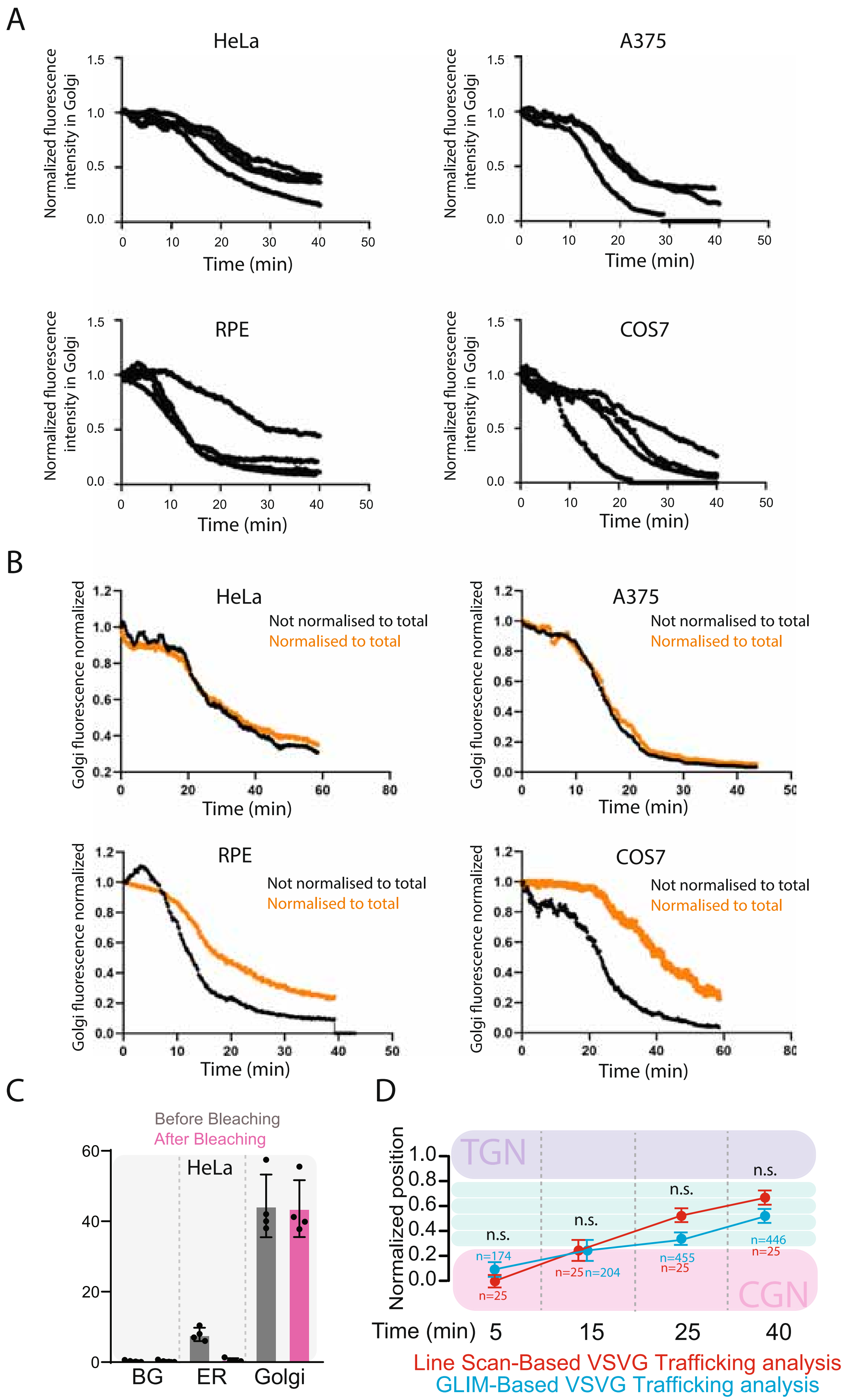
A
HeLa
A375
RPE
COS7
Normalized fluorescence intensity in Golgi
Time (min)
B
HeLa
A375
RPE
COS7
Not normalised to total
Normalised to total
Golgi fluorescence normalized
Time (min)
C
Before Bleaching
After Bleaching
HeLa
BG
ER
Golgi
D
TGN
CGN
Normalized position
n.s.
n=174
n=25
n=204
n=455
n=446
Time (min) 5 15 25 40
Line Scan-Based VSVG Trafficking analysis
GLIM-Based VSVG Trafficking analysis

© The Author(s)

Figure EV2. iFRAP analyses of cargo exit from the Golgi apparatus.

(**A**) The individual traces of the iFRAP data presented in Fig. 1B are shown. (**B**) Representative traces from A were normalized in two different ways. As presented in (**A**) or they were normalized by total fluorescence to account for bleaching (orange). The curves were qualitatively similar with flat phase followed by an exponential decrease. (**C**) The intensity levels of background (BG; area with no cells), ER and Golgi were measured before and after bleaching in the iFRAP protocol. The bleaching was efficient in the ER while there was little to no change in the Golgi fluorescence (mean ± SD; $n > 3$). (**D**) VSVG-GFP-RUSH construct localization with Golgi markers (GM130 and TGN46) from the replicate 2 of Fig. 2B. The positions of VSVG-GFP-RUSH and Golgi marker peaks were normalized, setting GM130 as 0 and TGN46 as 1. Measurements obtained using line scan analysis (red) are compared with those from the GLIM based automated method (blue) within the same experiment. Each data point represents an individual measurement, with results from both methods shown side-by-side for direct comparison. The relative position of the VSVG-GFP-RUSH construct is plotted at each indicated time point (median ± SE; *n* is indicated in the figure, n.s = not significant; [Student's *t* test]). Source data are available online for this figure.

© The Author(s)

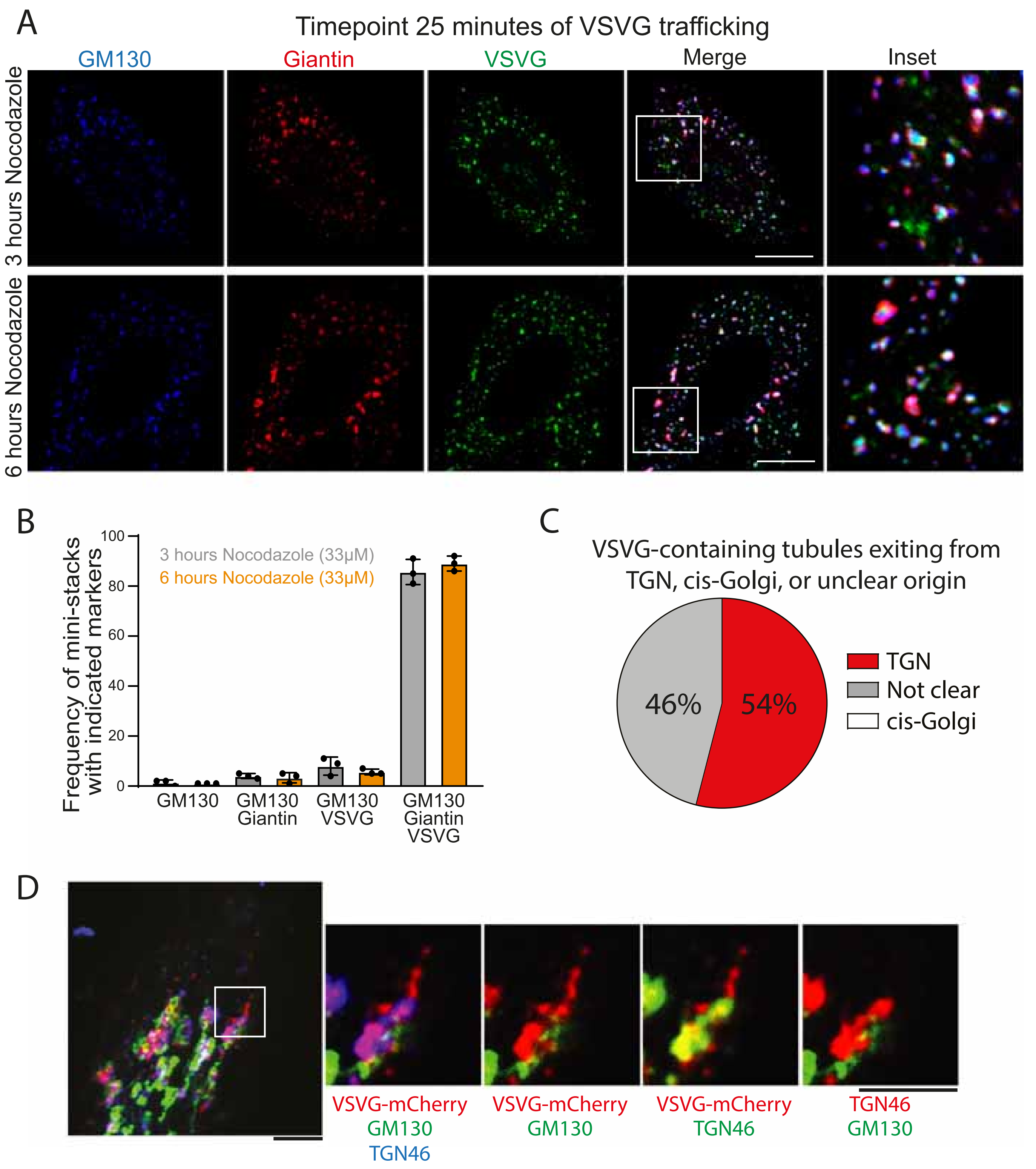
A
Timepoint 25 minutes of VSVG trafficking
GM130
Giantin
VSVG
Merge
Inset
3 hours Nocodazole
6 hours Nocodazole
B
Frequency of mini-stacks with indicated markers
100
80
60
40
20
0
3 hours Nocodazole (33μM)
6 hours Nocodazole (33μM)
GM130
GM130 Giantin
GM130 VSVG
GM130 Giantin VSVG
C
VSVG-containing tubules exiting from TGN, cis-Golgi, or unclear origin
46%
54%
TGN
Not clear
cis-Golgi
D
VSVG-mCherry GM130 TGN46
VSVG-mCherry GM130
VSVG-mCherry TGN46
TGN46 GM130

 © The Author(s)

Figure EV3. Validity of nocodazole treatment and exit of cargoes from the TGN.

(**A**) HeLa cells were transfected with VSVG-GFP and kept overnight at 40 °C. Then cells were treated with nocodazole for the indicated amount of time and shifted to 32 °C for 25 min and then fixed and prepared for immunofluorescence with the indicated markers. White boxes represent insets. Bar: 10 µm. (**B**) Quantification from (**A**) of the number of ministacks showing the indicated markers. Mean $+/-$ SD. Data points (n > 290) from 3 independent experiments. (**C**) The Pie chart shows the percentage of VSVG-mCherry-containing tubules exiting from TGN and those whose origin is unclear ($n = 13$). We have not observed any tubule originating from *cis*-Golgi. (**D** HeLa cells were transfected with VSVG-mCherry-RUSH construct overnight and then biotin was added for 1 h. The cells were fixed and stained for GM130 and TGN46 to mark the *cis*-Golgi and TGN respectively. White boxes represent insets. Bar: 5 µm. Source data are available online for this figure.

© The Author(s)

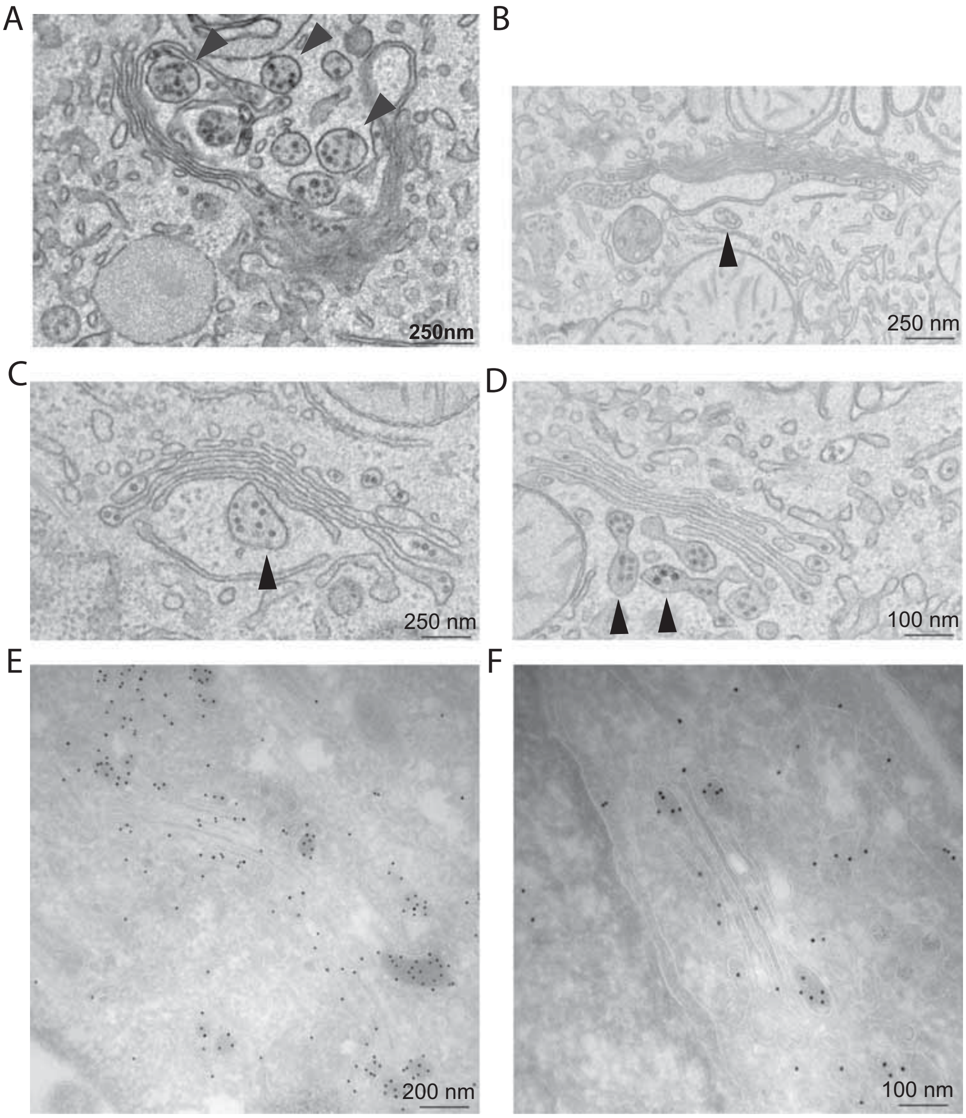

Figure EV4. Cargoes accumulate in the TGN before exiting the Golgi apparatus.

(**A**) Accumulation of VLDL particles in the TGN area of the hepatic tissue is indicated by black arrowheads. Bar: 250 nm. (**B–D**) Samples images showing the accumulation of VLDL particles in the TGN area of the hepatic tissue (indicated by black arrowheads as in Fig. EV4A). (**E**, **F**) Sample images from the same experiment of Fig. EV4A. Gold particles indicate GFP-FM4-hGH. Source data are available online for this figure.

 © The Author(s)
